# Exploring user experience and technology acceptance for a fall prevention system: results from a randomized clinical trial and a living lab

**DOI:** 10.1186/s11556-016-0165-z

**Published:** 2016-06-10

**Authors:** Daryoush D. Vaziri, Konstantin Aal, Corinna Ogonowski, Thomas Von Rekowski, Michael Kroll, Hannah R. Marston, Rakel Poveda, Yves J. Gschwind, Kim Delbaere, Rainer Wieching, Volker Wulf

**Affiliations:** Institute for Information Systems, University of Siegen, Kohlbettstr. 15, 57072 Siegen, Germany; Institute of Movement and Sport Gerontology, German Sport University Cologne, Am Sportpark Muengersdorf 6, 50933 Cologne, Germany; Institute of Biomechanics of Valencia, University Polytechnic of Valencia, Edificio 9C Camino de Vera s/n, 46022 Valencia, Spain; Neuroscience Research Australia, University of New South Wales, Barker Street, Randwick, Sydney, 2031 New South Wales, Australia

**Keywords:** Fall prevention, Game design, Exergames, Older adults, Usability, User experience, Technology acceptance

## Abstract

**Background:**

Falls are common in older adults and can result in serious injuries. Due to demographic changes, falls and related healthcare costs are likely to increase over the next years. Participation and motivation of older adults in fall prevention measures remain a challenge. The iStoppFalls project developed an information and communication technology (ICT)-based system for older adults to use at home in order to reduce common fall risk factors such as impaired balance and muscle weakness. The system aims at increasing older adults’ motivation to participate in ICT-based fall prevention measures. This article reports on usability, user-experience and user-acceptance aspects affecting the use of the iStoppFalls system by older adults.

**Methods:**

In the course of a 16-week international multicenter study, 153 community-dwelling older adults aged 65+ participated in the iStoppFalls randomized controlled trial, of which half used the system in their home to exercise and assess their risk of falling. During the study, 60 participants completed questionnaires regarding the usability, user experience and user acceptance of the iStoppFalls system. Usability was measured with the System Usability Scale (SUS). For user experience the Physical Activity Enjoyment Scale (PACES) was applied. User acceptance was assessed with the Dynamic Acceptance Model for the Re-evaluation of Technologies (DART). To collect more detailed data on usability, user experience and user acceptance, additional qualitative interviews and observations were conducted with participants.

**Results:**

Participants evaluated the usability of the system with an overall score of 62 (Standard Deviation, SD 15.58) out of 100, which suggests good usability. Most users enjoyed the iStoppFalls games and assessments, as shown by the overall PACES score of 31 (SD 8.03). With a score of 0.87 (SD 0.26), user acceptance results showed that participants accepted the iStoppFalls system for use in their own home. Interview data suggested that certain factors such as motivation, complexity or graphical design were different for gender and age.

**Conclusions:**

The results suggest that the iStoppFalls system has good usability, user experience and user acceptance. It will be important to take these along with factors such as motivation, gender and age into consideration when designing and further developing ICT-based fall prevention systems.

## Background

Digital gaming by and for older adults has become a popular area of research within the wider field of game studies and gerontology. There is an increasing awareness in medical and gerontology research that digital games, especially whole-body movement games, have the potential to reduce falls and increase overall health and quality of life (QoL) [1, 2]. Information and communication technology (ICT)-based programs offer a promising alternative method to reduce fall risk factors in older adults in their own home, using digital game-based systems and unobtrusive sensoring [3]. The main advantage is that these serious games for health, exergames in particular, can combine exercise and entertainment for older adults [4].

Long-term motivation and sustainable usage of exercise and health games by older adults remains unclear [5]. The factors which influence whether older adults accept or reject technologies in their lives are complex and diverse, include factors like gender and age [6–8], and differ greatly from younger technology users [9]. It is important to understand the technological requirements of older adults when designing high-quality systems [10, 11]. Ideally, older adults should be involved as “*co-designers*” in the process [12] to enable more detailed insights on day-to-day life aspects and interactions with digital technologies ([11] p.1199). This allows for the best possible approach to negotiate between design and user needs.

In order to successfully design such ICT-based systems for older adults, it is crucial to understand the users’ values, behaviours, attitudes, practices and technical experiences [13] regarding digital technologies and games. Usability, user experience and user acceptance play an important role in order to collect and interpret values, behaviours and practices of users for the design of such ICT-based systems. Using a combination of qualitative and quantitative data material, this paper aims to identify factors influencing usability, user experience and user acceptance of older adults engaging with an ICT-based fall prevention system (iStoppFalls).

## Methods

### Study setup

This paper is using data from an international, multicenter study designed as a single-blinded, two-group randomized clinical trial (RCT) with a total of 153 community-dwelling older adults (*n* = 78 intervention group, *n* = 75 control group) aged 65+ from Cologne, Germany (*n* = 59), Valencia, Spain (*n* = 37) and Sydney, Australia (*n* = 57) [3]; and a more qualitative-oriented Living Lab (LL) study with a total of 15 community-dwelling older adults aged 65+ from Siegen, Germany.

The iStoppFalls system used in this study consists of several technical components: 1) a set-top box with controller, 2) a mini personal computer (PC) with exergames, 3) a Microsoft-Kinect for movement detection, gesture and voice control, 4) a Senior Mobility Monitor (SMM) for mobility and activity tracking worn around the neck, 5) a tablet PC as an alternative input and output device for the interactive television (iTV) system, and 6) an iTV. A more detailed description can be found in [14].

In the RCT, the system’s effectiveness in terms of fall risk reduction and supplementary facets such as physical, cognitive and health related variables [15] were quantitatively analysed. The LL [16] qualitatively examined the systems’ suitability for integration into daily routines of older adults’, aspects of usability, user experience and user acceptance. Both, the RCT and LL, were conducted simultaneously. RCT group participants conducted a 16-week exercise program using the iStoppFalls system through the TV set in their own home [14]. LL participants conducted the same exercise program and setup for 24 weeks. The extended study period of the latter was based on the LL approach which requires long-term evaluation of qualitative aspects such as the integration into daily routines [17, 18]. Nevertheless, all participants used the exact same system including exergames and assessments.

### Study protocol

Predefined inclusion and exclusion criteria were applied to screen subjects [15]. Ethical approval was granted by the ethics committees of the German Sport University Cologne, the Polytechnic University of Valencia and the Human Research Ethics Committee of the University of New South Wales. The most relevant requirements for participation were a broadband internet connection, a high definition TVwith HDMI port and at least three meters space in front of the TV. For the LL study, an interest in preventive training (a weekly balance and strength training together with a monthly fall risk assessment) and the willingness to attend assessments or workshops at the different study centres had to be expressed. No financial compensation was offered to participants.

### Data collection

Data from different paper-based questionnaires relating to usability, user experience and technology acceptance were collected during the respective study periods. Feasibility of the questionnaires was pretested with 10 participants and revisions were conducted according to participants’ feedback. All questionnaires were distributed among the RCT and LL participants (at week 4, week 8 and week 16). In addition to quantitative questionnaires, qualitative data was collected by conducting face-to-face semi-structured interviews with a more detailed focus on usability, user experience and user acceptance. These interviews lasted between 30 and 120 min. For LL participants, face-to-face interviews were supplemented by observations regarding the interaction behaviour of participants with the iStoppFalls system and workshops with participants providing a platform to exchange and discuss ideas concerning the system.

### Measures

In total, 60 participants completed paper-based questionnaires (50 RCT and 10 LL). The sample constisted of 23 male and 37 female participants with an average age of 73 years. Table 1 provides an overview of participants’ characteristics.

**Table 1:** Participants’ characteristics

	Cologne	Valencia	Sydney	Siegen	Overall
Participants (n)	*n* = 15	*n* = 20	*n* = 15	*n* =10	*n* = 60
Intervention period (months)	4	4	4	6	4 to 6
Mean age (years, SD)	72.1 ± 3.6	71.5 ± 3.8	76.5 ± 4.6	70.9 ± 3.9	72.6 ± 4.0
Female (n, %)	9 (60)	13 (65)	9 (60)	6 (60)	37 (61,7)

*n* = number of participants, separated for each study center; Intervention period is presented in months, separated for each study center; Mean age is presented in years and standard deviation, separated for each study center; Females are presented with number of females and percentage of females, proportional to the total number of participants

The System Usability Scale (SUS) measures the usability of a product and consists of 10 items which are evaluated on a 5-point Likert scale ranging from 1 “strongly disagree” to 5 “strongly agree”. The results are distributed on a specific scale ranging from 0 for “worst imaginable” to 100 for “best imaginable” [19]. SUS is an appropriate and robust usability measure with easy application for the user [20, 21]. It is frequently applied in design studies evaluating the application of interfaces [22]. Recent publications illustrate a meaningful application of the SUS in evaluation settings with older adults and within a fall prevention context [23, 24].

The Physical Activity Enjoyment Scale (PACES) is frequently applied to measure the enjoyment of physical activities. Since physical activity is a core feature of the iStoppFalls system, the PACES was deemed appropriate to measure user experience. In this study, the 8-item PACES version was applied [25, 26]. All items are evaluated on a 6-point Likert scale. The responses from each participant are added up and averaged. A high score implies high enjoyment while being phyiscally active. The maximum enjoyment score for this scale is 48.

The Dynamic Acceptance Model for the Re-evaluation of Technologies (DART) is an instrument to analyze and evaluate the user acceptance of products or services without statistical analyses [27]. It provides a meta structure with four dimensions: perceived usefulness, perceived ease of use, perceived network effects and perceived costs. For each dimension individual acceptance indicators (AI) such as ease of use or attractiveness of design are defined to measure the importance and implementation perceptions of users in regard to the tested system. The definition of AI’s is based on literature review and user research in the field of ICT for older adults [28]. The users’ perceived importance to an AI and the users’ perceived implementation of an AI within the system are rated on a 6-point Likert scale for each indicator, with 1 “being very unimportant/totally unfulfilled” and 6 “being very important/totally fulfilled”. For each AI all ratings are averaged. Figure 3 in the results section illustrates the described method via spider charts using one line for the importance and one line for the implementation. Subtracting the importance ratings from the implementation ratings provide the degree of discrepancy between the users’ perception of importance and implementation within the tested system. This calculation gives a measure to explain the fulfillment of user expectations by the tested system. Values close to zero represent a high rate of acceptance from a users’ perspective.

### Interviews

Additional semi-strucutured interviews with a focus on health, mobility, system and technology use were conducted with 40 participants. We used open ended questions in order to obtain more detailed insights on participants’ perceptions. All interview participants were selected randomly, considering the predefined inclusion and exclusion criteria described above.

### Statistical analysis

A combination of qualitative and quantitative methods was used [29]. Descriptive analyses were conducted in SPSS version 22. The SUS and PACES were applied for the whole iStoppFalls system, whilst the DART was seperately applied for those participants performing the exergames and wearing the SMM. Therefore, DART data will also be presented for the exergame and SMM. All data was structured into two different perspectives, namely age (mean = 72) and gender.

Qualitative data analyses were conducted to further enhance our understanding of users’ attitudes and practices. Qualitative data analysis used a content analytic approach using MaxQDA version 12 [30]. Coding and codes which were formed based on the interview guides were supplemented by an open inductive coding based on the overall material [31]. Coding was undertaken by researchers who worked closely with the participants and were present during the intervention at the LL. They were supported by additional researchers who were not part of the LL. Empirical data was analyzed in respect of substantive data, coded interviews, transcripts, usability tests and workshops which were triangulated with additional observation notes taken right after every visit.

## Results

### Usability

**Fig. 1:**
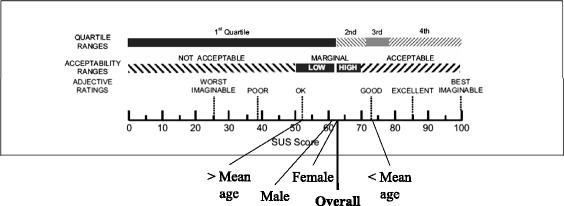
Overall System Usability Scale score. The SUS score is located on a scale ranging from 0 to 100. Adjective ratings provide interpretation of the SUS score. The acceptability range indicates whether the evaluated interface of the system is acceptable or not. Quartile ranges illustrate the average SUS score of all SUS studies (average SUS score of all SUS studies lies around 68), meaning that values around the 2^nd^ quartile represent an average result, based on all SUS studies

Participants evaluated the iStoppFalls system’s usability with an overall score of 62 (SD 15,58) which is good (See Fig. 1). In terms of gender, there was no noticable difference for the perception of usability. Male participants evaluated the usability of the system with a score of 61 (SD 19,17), while female participants evaluated the usability with a score of 62 (SD 23,46). In regard to participants’ age, our results illustrate that participants younger than the mean age (72 years) assessed the system’s usability with a score of 72 (SD 16,22) while participants older than the mean age assessed the usability with a score of 53 (SD 23,64). Figure 1 provides an overview of the SUS results.

During the interviews, participants reported technical issues with the system: *“In the beginning there were situations (…) I could have thrown the computer against the wall (…) but then, after new software has been uploaded, it worked much better, and I enjoyed playing the games again.”* (Siegen, Living Lab, 64 years, female).

Apart from the technical issues participants also stated that they perceived the system as user-friendly: “*The system, in its current state, can almost be described as ‘consumer-friendly’; especially for older adults. You always have to start from the premise that, especially older adults, struggle learning new technology - not concerning the exercises but rather pertaining to all the button pressing*.” (Siegen, Living Lab, 67 years, male). However, some older participants noted that the system was too complex to use without any help: “*However, when I was left alone in home to use it, I realised that there were many more things than what I was able to remember. I found it a bit overwhelming, and I was afraid of not using it right. I appreciated the instructions that helped me to go through the games.”* (Valencia, RCT, 73 years, male).

### User experience

**Fig. 2:**
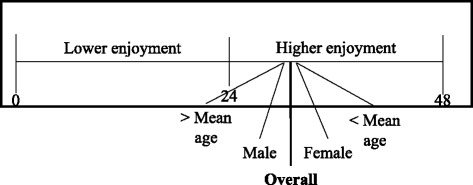
Overall Physical Activity Enjoyment Scale score. The scale ranges from 0 to 48. PACES scores below 24 are interpreted as lower enjoyment while being physically active. PACES scores above 24 are considered as higher enjoyment while being physically active

The overall enjoyment of using the iStoppFalls system was evaluated with a score of 31 (SD 8,03), which means enjoyable (see Fig. 2). In regard to gender, male participants evaluated the enjoyment of using the system with a score of 29 (SD 7,26), while their female counterparts evaluated the enjoyment with a score of 32 (SD 8,44). Younger participants (<72 years) assessed the enjoyment of using iStoppFalls with a score of 32 (SD 7,98). Older participants (>72 years) assessed the enjoyment with a score of 29 (SD 7,90). Figure 2 presents the PACES results.

The qualitative content analysis showed that participants’ enjoyment using the system was quite distinct. Some participants enjoyed playing the games: *„Overall I enjoyed the games, even though they were quite challenging and the Kinect didn’t work properly sometimes.“*(Siegen, Living Lab, 77 years, female). Over time, other participants found the games to become boring: *“Actually, I didn’t enjoy the games after some time. It just repeats over and over and gets a little boring then. If there would be an increase in the training (difficulty) then I could do it this way.”* (Siegen, Living Lab, 74 years, male).

### User acceptance

**Fig. 3:**
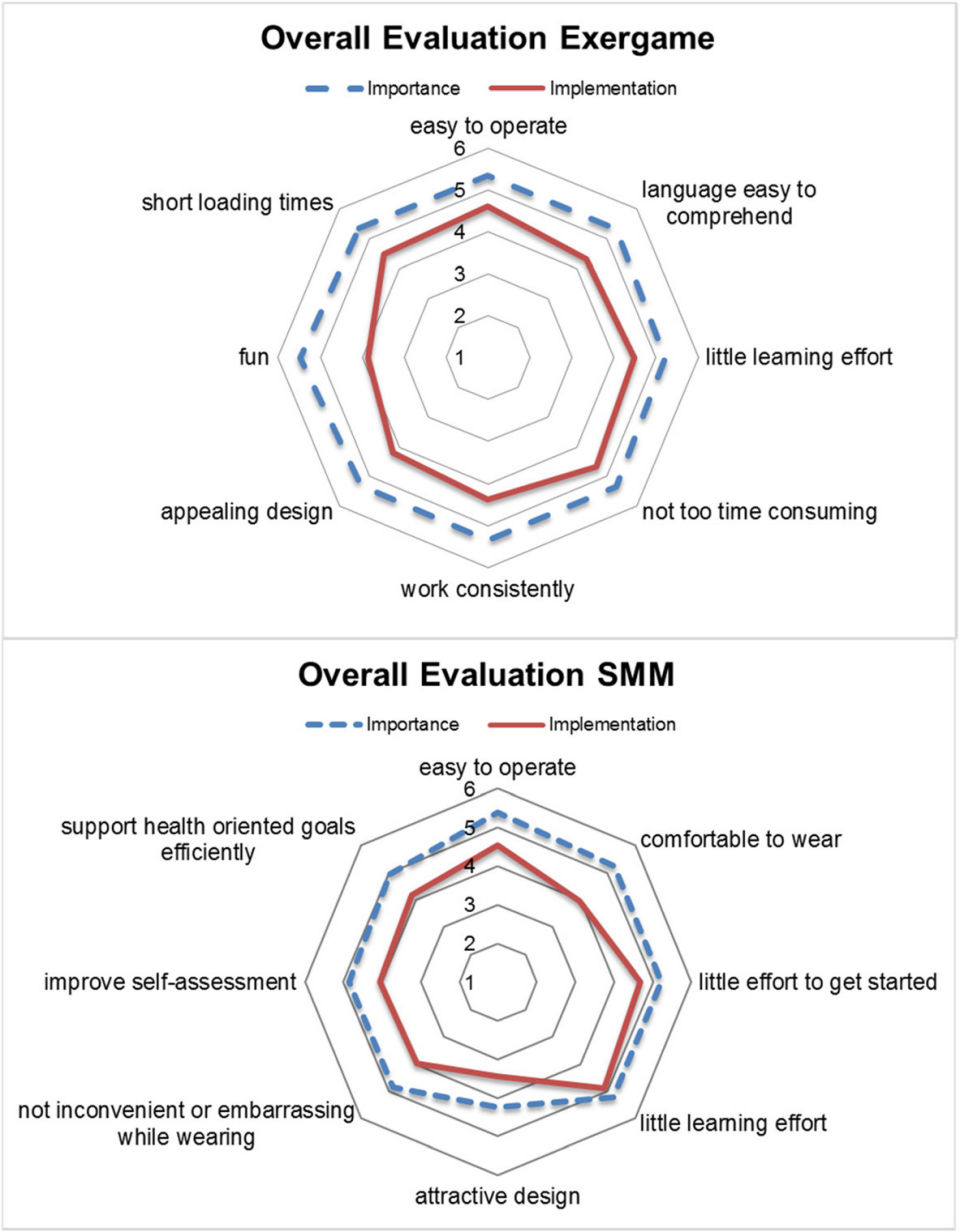
Overall evaluations for exergame and SMM (mean values). The dotted line represents the importance ratings for the acceptance indicators, while the solid line represents the implementation ratings for the acceptance indicators. Importance and implementation of all indicators are evaluated on a 6-point likert scale

The participants’ evaluation of the acceptance of iStoppFalls is illustrated in Fig. 3. The overall acceptance rate of the iStoppFalls system (SMM and exergame) was 0.87 (SD 0,26), which indicates a good user acceptance. Considering the exergame and SMM separately, the acceptance rate for the exergame was evaluated with a score of 0.96 (SD 0,28), whereas the acceptance rate for the SMM was rated with a score of 0.78 (SD 0,27). In terms of age, evaluations of participants younger than the mean age of 72 years resulted in an acceptance rate for the exergame of 0.44 (SD 0,18). Older participants’ evaluations resulted in an acceptance rate of 0.36 (SD 0.40). For the SMM, the acceptance rate evaluated by younger participants was 0.78 (SD 0,51), while older participants evaluated the acceptance for the SMM with 0.04 (SD 0,29). In regard to gender, male participants rated the acceptance of the exergame with 0.92 (SD 0.54), while female participants’ acceptance rate was 1.01 (SD 0.22). The acceptance of the SMM was 0.65 (SD 0.30) for male participants and 0.87 (SD 0.43) for female participants.

In addition, the interviews revealed specific factors influencing the acceptance of the system. For instance, some participants did not like the appearance of the SMM: “*Well, it [the SMM] just does not look good. Others may think - what does she have?! And the lights as well! I used it once in my gymnastics group and immediately turned the lamp to the body to disguise it.”* (Valencia, RCT, 73 years, female).

However, other participants reported liking the visualization of results: *“The pendant [SMM] was something that I wore in the beginning just because you told me: I wanted to contribute to your study, but I didn’t see the motivation of wearing that thing, until I saw the graphs with the results, the map, and so on. It was funny to see, and even tried to walk more outside to see if I could beat my record.”* (Valencia, RCT, 70 years, male). With regard to intentions to use the system after the study a participant stated: *Yes, I could imagine to keep on using the system for three times a week. But the system has to be improved. Technical failures occur too frequently and loading times are too long.”* (Cologne, RCT, 79 years, male). In terms of the exergame participants mainly responded in a positive way, aside from some technical issues: “*I really liked the variety of the games, especially the different forms of sport. The games were presented in a beautiful graphic. What I really liked on top of that was the ranking of results achieved while playing the games. I really got ambitious here. I couldn’t imagine becoming so ambitious about that before the study.”* (Cologne, RCT, 68 years, female).

## Discussion

In this paper, data from a home-based study on ICT-based fall prevention across different countries were investigated on usability, user experience and user acceptance. For the iStoppFalls system, age seemed to be an important factor for the perception and evaluation of the system’s usability. In respect to user experience, the findings suggest that game design should consider gender differences in order to make the use of such systems enjoyable for male and female users. Regarding user acceptance results, the study identified activity tracking devices like the applied SMM as an important component, because it may considerably motivate older adults to continuously use the system and be more active.

### Usability

Overall, participants perceived the usability of the iStoppFalls system as “good”. In respect to gender, there were no noticable differences in the evaluation of the system’s usability. However, regarding age, younger participants assessed the usability of the system better than older participants. As previously noted by participants during the interviews, malfunctions, long loading times or complex tasks prevented the appropriate use of the system. Referring to the results in our sample it seems that such usability issues had a stronger impact on older participants than on younger participants. These findings correspond to research investigating usability of ICT for older adults. [32] mentioned the importance of reducing malfunctions and providing easy solutions for older adults. In a different study, [33] found that complexity is a crucial factor regarding usability for older adults and should be reduced as much as possible.

### User experience

In general, the participants enjoyed using the iStoppFalls system. The results do not show any noticable differences for the enjoyment of the system regarding gender and age. However, the interviews revealed that there seems to be a gender difference regarding the enjoyment of the system. Some male participants stated that they found the games unchallenging or that the games became boring after some time. Such statements were not made by female participants, indicating that the user experience for male and female participants differs in the case of ICT-based fall prevention systems like iStoppFalls. This coincides with the current literature on gender differences in user experience of video games [8, 34]. Age differences with regard to user experience were not found in the qualitative data material. Considering gender differences for the design of exergames therefore seems to be an important factor in order to ensure good user experience for the target group.

### User acceptance

In terms of user acceptance our quantitative results showed that the iStoppFalls system was generally accepted by the target group, as all acceptance rates were close to zero. However, Fig. 3 shows some indicators for improvements of the tested prototype. The most noticable descrepancies between participants’ importance and implementation evaluation were: (1) the fun factor in exergames and (2) the wearing comfort of the SMM. With regard to age and gender, there were no additional noticable differences.

The qualitative analysis was able to depict a more detailed and distinct picture of user acceptance for the exergame and SMM.

According to the interview statements participants were satisfied with the exergames. Apart from some technical issues critizised by most participants, visual aspects such as the exergame graphics seemed to be an important aspect to young older participants. [35] reported similar findings in their study, suggesting that graphical design should be considered as a relevant acceptance factor for older adults. Fun in exergames might be an important factor as well, as reported by the participants during the interviews. [36] investigated the importance of fun in exergames for older adults. In the respective study, the researchers ascertained fun as a major factor for older adults in terms of exergames. They also revealed that when exergames were fun, older adults were more likely to replay the exergames than younger people [36].

In regard to the SMM, participants pointed out that they disliked the design of the device. They described it as appearing old and dated. Some participants even stated that they would try to avoid using it in public for that reason. Male participants in particular mentioned that they did not like wearing the SMM device like a necklace. These findings indicate that visual design and novelty of ICT technologies seem to be important acceptance factors. [37] found the desire to stay up-to-date as one of the most important factors for acceptance in his study. According to our analysis, this might be also relevant in the case of ICT-based fall prevention, since the SMM device (research prototype) did not seem to provide a “being up-to-date feeling” for study participants, due to its appearance. Besides feeling up-to-date the analysis indicated that gender specific aspects (wearing the SMM like a necklace) might have had an influence on the acceptance of the SMM as well. On the other hand, participants were very keen on the functionalities of the SMM device such as visualizing results based on tracked movement data. Such functionalities enabled participants to follow their own physical development and compare their results and achievements to others. These functionalities were mentioned as being very motivating by the participants. Studies investigating motivational aspects for the use of technology for physical activity by older adults illustrate that tracking and feedback of physical activity are important factors for that target group [38–40].

### Design implications

The analysis, presented in this article, showed that the design of ICT-based fall prevention systems for older adults should consider specific aspects with regard to usability, user experience and user acceptance. In general our quantitative and qualitative analysis revealed following design implications for exergames and activity trackers listed in Table 2.

**Table 2:** Design implications

No.	Exergames
1	Exergames should be easy to operate
2	The used language within the exergames should be easy to comprehend
3	It should require little learning effort to start using the exergames
4	It should not require much time effort to use the exergames
5	The exergames should work without malfunctions and have short loading times
6	Visualizations and graphical design should be attractive to the target group, especially for the young older adults
7	Playing the exergames should be fun for the target group
8	Exergames should provide different difficulty levels
	Activity trackers
9	Activity trackers should be easy to operate
10	Wearing such devices should be comfortable and convenient with regard to gender specific preferences
11	The effort required to start using such devices needs to be little
12	The effort to learn how to use such devices should be little
13	The design of activity trackers should be attractive to the target group
14	Activity trackers should provide visualizations for results to enhance user motivation

Design implications are separated in the categories “exergames” and “activity trackers”. Each design implications has a preceded identification number

In addition to the design implications summarized in Table 2, observations and qualitative interviews revealed that graphical and fun aspects of exergames are more important for younger old adults than older old adults. With regard to gender, the design of exergames may consider different contents. Combination with wearable devices like the SMM increases the motivation of older adults to use ICT-based fall prevention systems and therefore constitute an important factor for a sustainable design of fall prevention systems.

### Limitations

The questionnaires used in this study were applied in a way that the SUS and PACES covered the iStoppFalls system as a whole, while the DART differentiated between exergame and SMM allowing participants to make more distinct and accurate evaluations of system components. The SUS and PACES results therefore do not give insight into how usability and user experience were perceived for the exergame or the SMM. However, our results provided valuable qualitative information about usability and user experience aspects from participants in regard to the exergame and the SMM. Finally, the sample size of this study was too small to allow for detailed statistical analyses in terms of user acceptance. Therefore, our results can not provide significances or correlations within the collected data set.

## Conclusions

The results suggest that the iStoppFalls system has good usability, user experience and user acceptance and discuss the importance of taking into account age and gender. This paper provides important information regarding motivational and sustainable use aspects, which can be used when designing usable and enjoyable ICT-based fall prevention systems. In order to achieve sustainable system use, it will be important to consider these aspects and providing motivational factors will facilitate the acceptance of ICT-based fall prevention systems by the target group.
